# Potential for Increased Human Foodborne Exposure to PCDD/F When Recycling Sewage Sludge on Agricultural Land

**DOI:** 10.1289/ehp.6802

**Published:** 2004-04-26

**Authors:** Karen Rideout, Kay Teschke

**Affiliations:** ^1^School of Occupational and Environmental Hygiene; ^2^Institute for Resources, Environment and Sustainability; ^3^Department of Health Care and Epidemiology, University of British Columbia, Vancouver, British Columbia, Canada

**Keywords:** agriculture, bioaccumulation, biosolids, dioxins, exposure assessment, food chain, furans, land recycling, PCDD/F, plant uptake, sewage sludge

## Abstract

Sewage sludge from municipal wastewater treatment is used in agriculture as a nutrient source and to aid in moisture retention. To examine the potential impact of sludge-amended soil on exposures to polychlorinated dibenzo-*p*-dioxins and dibenzofurans (PCDD/Fs) from plant and animal foods, we conducted a review of published empirical data from international sources. Levels of PCDD/F in municipal sewage sludge ranged from 0.0005 to 8,300 pg toxic equivalents (TEQ)/g. Background levels in soil ranged from 0.003 to 186 pg TEQ/g. In sludge-amended soils, levels of PCDD/F ranged from 1.4 to 15 pg TEQ/g. Studies that measured levels before and after sludge treatment showed an increase in soil concentration after treatment. Relationships between PCDD/F levels in soil and resulting concentrations in plants were very weakly positive for unpeeled root crops, leafy vegetables, tree fruits, hay, and herbs. Somewhat stronger relationships were observed for plants of the cucumber family. In all cases, large increases in soil concentration were required to achieve a measurable increase in plant contamination. A considerably stronger positive relationship was observed between PCDD/F in feed and resulting levels in cattle tissue, suggesting bioaccumulation. Although PCDD/Fs are excreted in milk, no association was found between feed contamination and levels of PCDD/Fs measured in milk. There is a paucity of realistic data describing the potential for entry of PCDD/Fs into the food supply via sewage sludge. Currently available data suggest that sewage sludge application to land used for most crops would not increase human exposure. However, the use of sludge on land used to graze animals appears likely to result in increased human exposure to PCDD/F.

In populations not industrially exposed to polychlorinated dibenzo-*p*-dioxins and dibenzofurans (PCDD/Fs), diet is responsible for virtually all (~ 98%) human exposure to these compounds (Pohl et al. 1995; Travis and Hattemer-Frey 1987). PCDD/Fs are common contaminants in municipal sewage sludge; thus, it is important to consider the risk of increased exposure to these contaminants if sewage sludge is to be applied to agricultural lands. There is currently much interest in agricultural use of sewage sludge to reap its benefits as fertilizer, as an aid in moisture retention, and to provide an alternative to incineration or landfills for disposal. The term “sewage sludge” is used here to refer to the solid by-product of municipal sewage or wastewater treatment processes. It includes but is not limited to “biosolids,” a term that usually refers to a stabilized product that has been treated to reduce pathogen content and vector attraction potential. The more inclusive term is used here because the data used in this review included all forms of municipal sewage sludge and because PCDD/F content is not affected by the additional treatment processes.

Some authors who have examined food-borne exposure to PCDD/F via sewage sludge have conducted deterministic modeling, using a number of assumptions including sludge application rates, exposure duration, PCDD/F concentration in sewage sludge, application methods, timing of application with respect to harvesting or sampling, and impact of atmospheric deposition. Those interested in such reports are referred to Duarte-Davidson and Jones (1996), Jackson and Eduljee (1994), Jones and Sewart (1997), Rappe and colleagues (1999), Wild and Jones (1992), and Wild and colleagues (1994). The U.S. Environmental Protection Agency (U.S. EPA) has recently modeled disease risks (cancer) from land-applied sewage sludge (U.S. EPA 2004).

In this article, we review the international empirical evidence of the impact of contaminated soil on the concentrations of PCDD/F in plant and animal tissue. We undertook this review to provide guidance regarding agricultural use of sewage sludge to federal, provincial, and municipal governments in Canada. Our purpose was to examine only the empirical literature and to use that literature to describe the potential transfer of PCDD/F from soil to foodstuffs, to derive empirical models of the transfer, and to identify data gaps in the science. We also wanted to determine whether some agricultural uses present greater likelihood than others of increased PCDD/F consumption by humans.

To organize the literature review process, we considered the pathways by which PCDD/F might be transferred from sewage products to humans via the food supply. Contaminants may adhere directly to plant surfaces or they may move from the sludge into the soil. From the soil, they may be transferred to crops, which are then consumed by humans or animals. These animals may in turn be consumed by humans. Animals also consume soil while grazing, which potentially increases their contaminant load.

## Methods

### Literature Search

A systematic search of the published literature was conducted using the following databases: MEDLINE (http://gateway2.ovid.com/), TOXLINE (http://toxnet.nlm.nih.gov/), Agricola (http://agricola.nal.usda.gov/), National Technical Information Service (http://www.ntis.gov/search/index.asp?loc=3-0-0), EMBASE (http://www.embase.com/), CAB International Abstracts (http://www.cabi.org/), Environmental Sciences and Pollution Management (http://ca1.csa.com), Food Science and Technology Abstracts (http://www.foodsciencecentral.com), Web of Science (http://isiknowledge.com), Compendex (http://www.engineeringvillage2.org), Dissertation Abstracts (http://wwwlib.umi.com/dissertations/gateway), Public Affairs Information Service, and Canadian Institute for Scientific and Technical Information (http://cat.cisti.nrc.ca/screens/opacmenu.html).

Combinations of the following key words were used in the searches: agricultural, agriculture, animals, application to land, application to soil, biosolids, crops, cropland, dibenzofuran, dioxin(s), fluid waste disposal, food contamination, forage, furan(s), land application, PCDD/F, PCDD, PCDF, plants, polychlorinated dibenzo-*p*-dioxin, polychlorinated dibenzofuran, sewage, sewage sludge, sewage as fertilizer, soil, soil ingestion, and soil pollutant.

In addition, literature previously gathered by the British Columbia Ministry of Water, Land and Air Protection was provided to us. Reference lists of all relevant articles including review articles were used as a source of additional citations.

Literature was sought in relation to the following issues: *a*) levels of PCDD/F in municipal sewage sludge; *b*) background levels of PCDD/F in soil; *c*) levels of PCDD/F in soil after sewage sludge application; *d*) transfer of PCDD/F from soil to plant tissue; *e*) transfer of PCDD/F from soil or feed to tissue of grazing animals.

### Inclusion and Exclusion Criteria

All articles identified by the search were reviewed for relevance using the title and/or abstract. Articles were considered relevant if they reported PCDD/F concentrations in the following sample types: sludge from sewage or wastewater treatment plants handling municipal wastes; agricultural soil with historical or experimental treatment with sewage sludge; agricultural soil with no previous application of sewage sludge or experimental contamination with PCDD/Fs; food or forage plants grown in sludge-amended soil or soil treated experimentally with PCDD/Fs; tissue or milk of animals fed food grown in sludge-amended soil or food otherwise contaminated with PCDD/F; tissue of animals grazing on sludge-amended soil; or plant food, forage crops, animal tissue, or milk not believed to be contaminated from a specific PCDD/F source, that is, background concentrations in these types of food.

The following types of publications were excluded from further review: those that were not peer reviewed; those that reported about sites of industrial accidents (e.g., Seveso, Italy), nonmunicipal sources of sludge (e.g., industrial waste, pulp mill sludge), or plants grown by soil-free methods (e.g., hydroponics); studies conducted before 1980 when the limits of analytical chemical methods were insufficient to detect low PCDD/F concentrations; or studies that used nonstandard analytical methods (e.g., bioassays to determine dioxin-like activity).

Sixty-five papers met the above criteria.

### Data Treatment and Analysis

All PCDD/F concentrations were converted to equivalent units using the international toxicity equivalency system (U.S. EPA 1999).

To examine the relative uptake of PCDD/Fs from soil to different plant and animal tissues, simple linear regressions were conducted to estimate the relationships between soil or feed PCDD/F toxic equivalents (TEQ) concentration (independent variable) and plant or animal tissue concentration (dependent variable) for each tissue type with a minimum of five data points. The resulting regression coefficients and standard errors were used to predict potential tissue PCDD/F concentrations (in TEQ) over the range of soil PCDD/F concentrations observed in agricultural settings where sewage sludge had been applied to the land. All analyses were performed using JMP statistical analysis software, version 3.2 (SAS Institute, Cary, NC).

## Results

### Sewage Sludge Contamination by PCDD/F

In municipal sewage sludge, levels of PCDD/F ranged from 0.0005 to 8,300 pg TEQ/g (Table 1).

### Soil Contamination by PCDD/F

**Figure 1:**
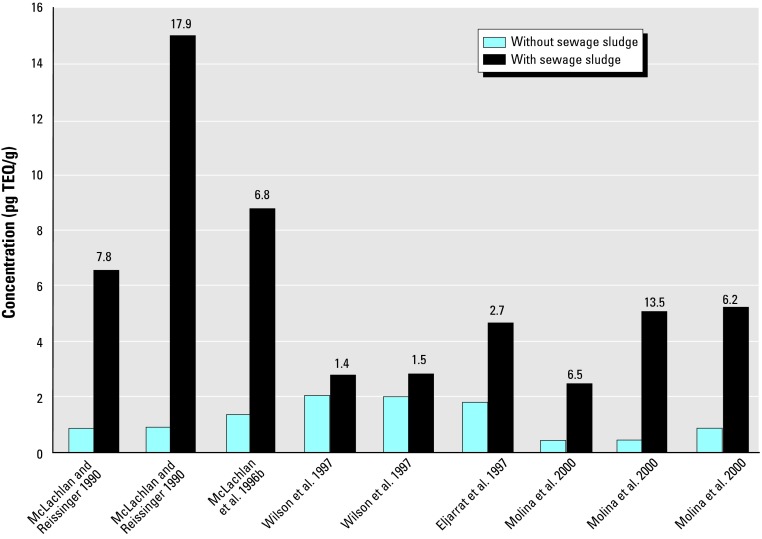
Change in concentration of PCDD/F in soil after sludge application. The numbers above the bars indicate the factor by which the soil PCDD/F concentration increased after application of sewage sludge.

Background levels of PCDD/F in untreated soils ranged from 0.003 to 186 pg TEQ/g (Table 2). In studies of soil after sludge application, concentrations of PCDD/F ranged from 1.4 to 15 pg TEQ/g (Table 2). Although this range is very similar to the range of background values in untreated soils, all studies that measured soil PCDD/F concentrations before and after sludge application found increased contamination after sludge amendment (Figure 1). PCDD/F concentrations increased by factors of 1.4 to 17.0 (mean 7.1) after sludge application, indicating that application of sewage sludge increases PCDD/F contamination in soil.

### Crop Contamination by PCDD/F

Table 3 is a list of the levels of PCDD/F in root crops, including carrots, potatoes, and beets. Mean levels in crops grown in uncontaminated soil or soil with low levels of PCDD/F ranged from below detection limits (<0.01) to 0.6 pg TEQ/g dry weight (dw).

Root vegetables grown either in naturally contaminated soil or soil to which PCDD/F had been added for experimental purposes had concentrations ranging from below detection limits (detection limit not stated) (Prinz et al. 1991) to 6,488 pg TEQ/g (dw) (Table 3). All experimental studies that examined root uptake of PCDD/F used soils that were much more highly contaminated than sludge-amended agricultural land. PCDD/F concentrations in experimentally contaminated soil ranged from 56 to 112,800 pg TEQ/g soil, whereas the highest level found in treated agricultural soil was 49 pg TEQ/g soil.

Table 4 indicates the levels of PCDD/F in crops with edible parts grown above the ground, including lettuce, silver beet, peas, and zucchini. The concentrations of PCDD/F in the aboveground parts of crops grown in soil with low levels of PCDD/F contamination ranged from < 0.01 to 10.2 pg TEQ/g (dw) (Table 4).

When grown in more highly contaminated soil, aboveground plants, including lettuce, silver beet, peas, zucchini, pumpkin, kale, chives, endive, leeks, beans, kohlrabi, and savoy, had PCDD/F concentrations ranging from 0.04 to 55.2 pg TEQ/g (dw) (Table 4). Tree fruits such as plums, strawberries, and apples had PCDD/F concentrations ranging from 0.8 to 1.4 pg TEQ/g (dw) when grown in soil containing 670 pg TEQ/g PCDD/F. Apples and pears grown in soil containing from 48 to 1,950 pg TEQ/g (dw) PCDD/F contained from 8 to 142 pg TEQ/g fresh weight (fw) PCDD/F (Table 5).

Measured concentrations of grasses and hay grown in soil with low levels of dioxin and furan contamination were all ≤ 1 pg TEQ/g (Table 6).

The contamination levels found in grass and hay grown in contaminated soil were generally higher (0.1–39 pg TEQ/g) (Table 6). Of the two studies that examined PCDD/F contamination of forage grown in contaminated soil, one did not state whether the plants were washed before analysis (Prinz et al. 1991), and the other used sand or clay pebbles on the soil surface to prevent soil–leaf contact (Hulster and Marschner 1993).

### Relationships between PCDD/F in Soil and Crops

**Figure 2:**
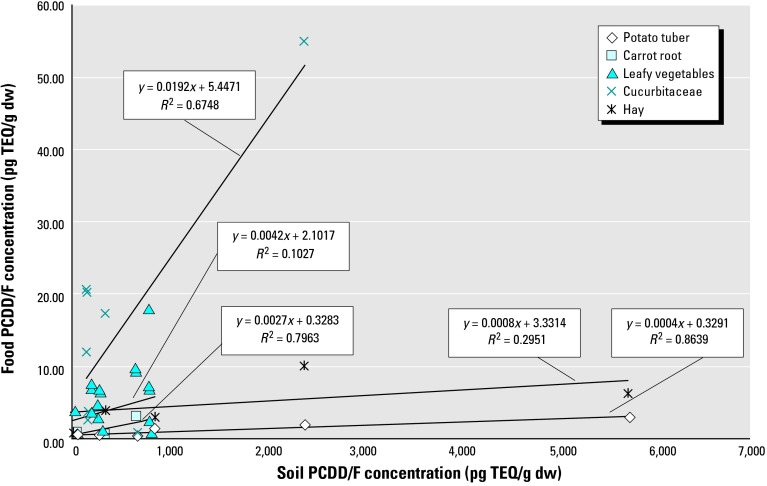
Relationship between PCDD/F concentrations in plant foods and soil contamination levels. The plant data include data from Tables 3, 4, and 6 that relate to those plants for which relationships could be found between plant and soil PCDD/F concentrations. The following data were omitted: *a*) measurements in which the soil PCDD/F concentration was much higher (8- and 20-fold) (Hulster and Marschner 1993) than in the other samples and not remotely relevant to the soil concentrations likely to result from sewage sludge application; and *b*) the result of a study that did not use natural growing conditions (plants growing in pots of uncontaminated soil placed in or on top of contaminated soil (Hulster et al. 1994). Data were taken from the following sources: potato: Prinz et al. (1991), Hulster and Marschner (1993); carrot: Prinz et al. (1991), Schroll and Scheunert (1993), Muller et al. (1994); leafy vegetable: Prinz et al. (1991), Hulster and Marschner (1993), Muller et al. (1994); Cucurbitaceae: Prinz et al. (1991), Hulster et al. (1994); hay: Hulster and Marschner (1993).

Tables 3–7 and Figure 2 show the relationship between PCDD/F concentrations in soil and resulting concentrations in crop tissues. The contaminant levels in whole carrot and potato showed weak positive relationships with the contaminant level of the soil. The concentration in peeled potatoes, however, did not change over a wide range of soil concentrations. This suggests that most of the PCDD/F contamination in potatoes accumulates in the peel.

A positive relationship was found between some members of the cucumber (Cucurbitaceae) family (namely zucchini, pumpkin, and cucumber) and soil contamination levels. Concentration of PCDD/F in green leafy vegetables also showed a positive (though weaker) relationship with soil concentration. Among aboveground crops, the weakest positive relationship was present between soil PCDD/F concentrations and contamination of tree fruits such as apples and pears. The data were insufficient to estimate the relationship between soil and plant concentrations of PCDD/F in peas and beans. Weak positive relationships were observed between soil and plant concentrations of hay and herbs. No positive relationship was observed between concentrations of PCDD/F in soil and grass (Figure 2; Table 7).

### Animal Food Contamination by PCDD/F

Background contamination of beef ranged from less than the detection limit to 30.8 pg TEQ/g fat (Table 8); all mean values were < 5 pg/g. Dairy products were contaminated in the range of 0.3–1.4 pg TEQ/g fat (Table 8). Unfortunately, the contamination level of the feed eaten by the animals tested in these studies is not known.

Tissue concentrations from cattle consuming feed contaminated with PCDD/Fs ranged from 0.6 to 130 pg TEQ/g, in such tissues as fat, liver, kidney, muscle, and plasma (Table 8). Cattle were fed food with an extremely wide range of PCDD/F concentrations, ranging from those typically expected from forage crops (e.g., 2–3 pg/g) to extremely high levels (equivalent to thousands of picograms per gram) higher than the levels observed in sludge. For example, Jones et al. (1989) fed cattle 0.05 μg 2,3,7,8-tetra-chlorodibenzo-*p*-dioxin (TCDD)/kg body weight, which corresponds to a dose of 24.4 × 10^6^ to 32.5 × 10^6^ pg. Based on an estimated daily dry feed intake of 8 kg for beef cattle (Jones and Sewart 1997), this dose represents a feed contamination level of approximately 3,050–4,063 pg (mean 3,557) TEQ/g (dw). In those studies that used feed grown on sludge-amended land (Jilg et al. 1992; McLachlan et al. 1990, 1994; McLachlan and Richter 1998; Richter and McLachlan 2001), it was not stated whether the plants were washed or otherwise treated to remove soil or sludge particles before analysis and feeding. In practice, it is highly unlikely that grass, hay, or other forage would be washed before feeding to animals.

One of the great difficulties facing those studying animal uptake and contamination is the long duration required for animals to reach steady-state body burdens. The elimination half-life of PCDD in lactating cows is estimated to be in the range of 50–76 days (Firestone et al. 1979; Tuinstra et al. 1992), although one study based on a large single dose of 2,3,7,8-TCDD found that most was excreted in the milk within 14 days (Jones et al. 1989). The biological half-life of PCDD/F in cattle has been estimated to be somewhat longer, on the order of 93–148 days (Jensen et al. 1981; Thorpe et al. 2001), based on two experiments in which the animals were fed for 28 days and 18 weeks. Furthermore, McLachlan et al. (1994) found higher PCDD/F concentrations in cows that had calved several times than in those that had calved only once, suggesting that steady state had not been achieved in the younger cows. The exposure time in most of the feeding studies found in the literature search ranged from a single dose to 19 weeks. Given that it takes about five biological half-lives to reach steady state, the estimated minimum time to reach steady state would be 250 days in lactating animals and 465 days for nonlactating animals. None of the feeding studies were of sufficient duration.

Concentrations of milk from cows consuming PCDD/F-contaminated feed ranged from 0.031 to 3.0 pg TEQ/g (Table 8). Cows were fed food with PCDD/F concentrations typically expected from forage crops (e.g., 0.3–3 pg/g). As in the animal tissue studies, none of the studies was of sufficient duration for the body burden to reach steady state, although because of the shorter PCDD/F half-life in lactating animals and a minimum feeding duration of 17 days, the milk studies were generally more realistic. It should be noted that in most of these studies, milk was sampled while contaminated feed was still being consumed (Fries et al. 1999; Jilg et al. 1992; McLachlan et al. 1990, 1994) or within a week after the contaminated feeding ceased (Jilg et al. 1992; McLachlan and Richter 1998).

Among those who studied PCDD/F levels in milk with differing levels of soil or feed contamination, two reported little or no effect (Furst et al. 1993; McLachlan and Richter 1998), although the latter study did observe a slight increase in whole milk PCDD/F concentrations from 0.015 pg TEQ/g before the intervention to 0.049 pg/g after 23 days of consuming feed contaminated with 3.2 pg TEQ/g. Fries et al. (1999) found a 17-fold increase in dioxin and furan contamination of milk fat after pentachlorophenol-treated wood (contaminated with PCDD/F) was added to the cow’s diet for 58 days. McLachlan et al. (1994) found that the application of sewage sludge as fertilizer for harvested feed can increase the PCDD/F concentration in milk under certain circumstances, that is, in cows with a low level of milk production or in cows lactating after their first calving.

### Relationships between PCDD/F in Feed and Animal Tissues

**Figure 3:**
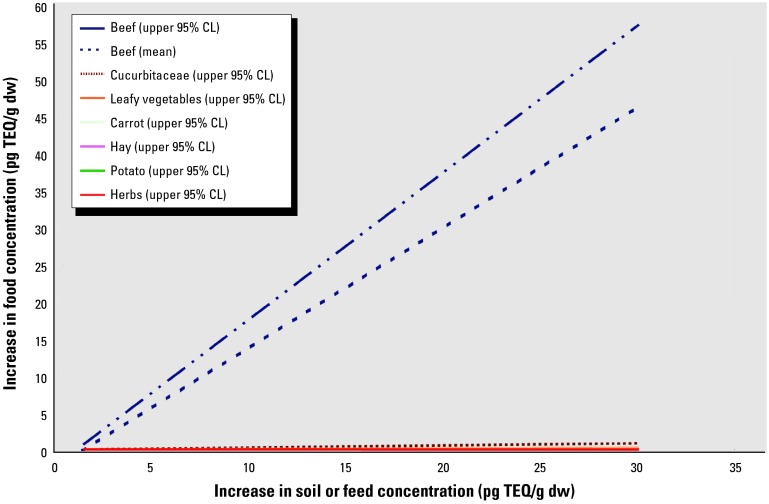
Projected increases in PCDD/F concentrations in plant foods and beef per unit increase in soil or feed contamination levels. CL, confidence limit.The data are derived from the regression curves for plant and animal foods shown in Table 7. This figure illustrates the increases in PCDD/F concentrations in beef fed feed or forage contaminated with PCDD/F and demonstrates how much more pronounced this effect is in beef than in the plant foods grown in sludge-treated soils. The regression curve for beef includes all values from Table 8 relating to concentration of PCDD/F in beef tissue (not milk) that provided the feed PCDD/F level (Jensen et al. 1981; Jilg et al. 1992; Richter and McLachlan 2001; Thorpe et al. 2001) except one study that used an experimental dose 87 times higher than in the other studies (Jones et al. 1989).

Table 8 and Figure 3 show the relationship between PCDD/F concentrations in feed and resulting concentrations in animal tissues. Because all results were reported per gram of lipid and there was no consistent pattern by tissue type, (i.e., muscle, fat, plasma, kidney, liver), all values were included in a single regression curve. The contaminant levels in beef tissue showed a strong positive relationship with the contaminant level in the feed.

No clear pattern was observed in the data from five studies examining the relationships between contamination of feed or grazing land and milk contamination from cows (Fries et al. 1999; Jilg et al. 1992; McLachlan et al. 1990, 1994; McLachlan and Richter 1998).

## Discussion

### Sewage Sludge and Soil

Soils treated with sewage sludge had relatively low levels of contamination when compared with those of the sludge itself. It is important to note, however, that in every case, the concentration of PCDD/F in the soil increased measurably after sludge application (Eljarrat et al. 1997; McLachlan and Reissinger 1990; McLachlan et al. 1996b; Molina et al. 2000; Wilson et al. 1997) (Figure 1). The elevated concentration of PCDD/F in sludge-amended soil also persisted over time. Most of the studies (Eljarrat et al. 1997; Molina et al. 2000; Wilson et al. 1997) measured PCDD/F concentrations up to 1 year after application of sewage sludge. One study that measured contamination on reclaimed quarry soil found elevated PCDD/F concentrations 4 years after a single treatment with sludge (Molina et al. 2000). Another study using archived soil samples from land that received a single sludge application in 1968 found that 59% of the PCDD/F contamination detected in 1972 was still present 18 years later (McLachlan et al. 1996b). McLachlan and Reissinger (1990) compared fields with 10–30 years of regular sludge treatments (application rate not known) with an untreated field on the same farm and found higher PCDD/F concentrations in the treated fields. Only one other study examined the effect of multiple sludge treatments (Eljarrat et al. 1997); after four annual treatments, the authors reported soil contamination levels no higher than those reported in other studies of single sludge treatments. In another study that compared the effects of plowing sewage sludge into the soil with surface application on meadowland, the authors found that elevated PCDD/F concentrations persisted for at least 260 days after application of sewage sludge and appeared to be slightly more persistent when plowed into the soil (Wilson et al. 1997). The half-life of PCDD/F in soil is estimated to be at least 10 years (Jackson and Eduljee 1994; Rappe et al. 1999).

### Plant Foods

Studies that examined the uptake of PCDD/F by plants growing in contaminated soils used either field soils that were highly contaminated because of proximity to heavy industry or experimentally contaminated soils with extremely high levels of PCDD/F. The PCDD/F concentrations in the soils used as controls in these studies are closer to if slightly lower than the concentrations found in sludge-amended agricultural soils. Furthermore, differences in soil properties, such as organic matter content, between contaminated and sludge-amended soils may affect plant uptake.

In our estimates of the relationships between soil and plant concentrations, the slopes of the regression lines were very shallow, suggesting that large increases in soil contamination would be required for small increases in plant contamination (Table 7, Figure 2). The regression coefficients and standard errors were used to estimate mean PCDD/F contamination levels in crops grown in soil with contamination levels in the range found for sludge-amended soils. These estimates indicate that very little change in plant contamination is expected over the probable soil contamination range of 1–30 pg TEQ/g soil. Even at an extremely high estimate for soil concentration, one that assumes a concentration equivalent to that of the highest sludge concentration reported, the predicted increases in plant concentrations were only moderately elevated. It is important to note, however, that the predicted plant values at the lower soil contamination levels have been back-extrapolated, as no empirical data are available at these lower soil concentrations. This adds uncertainty to the estimates.

Interpretation of the coefficients listed in Table 7 must take into account that they are based on relatively few data points, from only one or a few studies. Taken together, they suggest that for most plants, large increases in soil contamination (200–10,000 pg TEQ/g; namely, much higher than the increases expected from sewage sludge treatment) are required to produce small increases (1 pg TEQ/g) in plant contamination. They also suggest that plants in the family Cucurbitaceae (pumpkin, zucchini, cucumber) show a sufficiently strong association between soil PCDD/F levels and plant contamination that application of sewage sludge may increase the contamination levels of the plants.

The data suggest that different plants have different potentials for uptake of PCDD/Fs based on the different coefficients for the relationships between soil contamination levels and plant concentrations. All studies that examined the uptake of PCDD/Fs from soil by carrots and by certain members of the cucumber family found that these plants take up more PCDD/Fs from the soil than do other plants. In a study comparing different members of the family Cucurbitaceae (Hulster et al. 1994) grown in contaminated soil (148 pg TEQ/g soil), zucchini fruits and the outer layer of pumpkin (genus *Cucurbita*) had much higher levels of PCDD/F contamination [20.0 and 11.8 pg TEQ/g (dw), respectively] than did cucumber (genus *Cucumis*) [2.35 pg TEQ/g (dw)]. In a study that compared the ability of root exudates to absorb PCDD/F from soil (Hulster and Marschner 1994), zucchini root exudates absorbed 4 times more PCDD/F than tomato root exudates.

In a study that measured PCDD/F uptake by carrots grown in contaminated soil (Muller et al. 1994), more than 75% of the contamination was concentrated in the peel [mean concentration, 3 pg TEQ/g (dw)]. The inner parts of the carrot had PCDD/F concentrations more comparable to other plants [mean cortex concentration, 0.29 pg TEQ/g (dw); mean stele concentration, 0.40 pg TEQ/g (dw)]. When the congener profiles were compared, although the control (uncontaminated) soil had primarily octachlorodibenzo-*p*-dioxin (OCDD) and the contaminated soil had mostly higher chlorinated furans, the carrots from either soil contained mostly lower chlorinated furans. The lower-chlorinated PCDD/F congeners tend to be more bioavailable in lipid environments (Muller et al. 1993), which declines from the outer to inner parts of the carrot root.

Although the published empirical data for any one crop are very limited, the collective body of work indicates that high levels of PCDD/F in soil are associated with increased contamination of plant crops. However, at the soil contamination levels expected from treatment with sewage sludge, it appears that there would be minimal or no increase in the dioxin and furan content of most food crops.

To date, there is no evidence related to the potential for increased dioxin and furan contamination of other root vegetables (e.g., beets, parsnips, turnips, sweet potatoes, ginger, garlic, onions) or aboveground plant foods (e.g., cruciferous vegetables, berries, tomatoes, corn, peppers, grains).

### Forage Crops

Studies that have examined the uptake of PCDD/Fs by forage crops, such as the studies on other plant foods, used soils with extremely high levels of PCDD/F. Within this wide range of soil contamination levels, weak positive relationships were seen between soil and hay or herb concentrations of PCDD/F, but not between soil and grass concentrations. Potential contamination levels of hay and herbs grown on sludge-amended land were estimated using the regression coefficients (Table 7). Over the soil contamination range of 1–1,250 pg TEQ/g soil, there is virtually no change in predicted crop contamination levels.

Although the evidence for forage crops appears consistent with that of other plants with edible parts grown aboveground, there are outstanding issues relating to adherence of soil particles to the plants. In one study that measured the soil content of freshly cut forage from a pasture, the soil content ranged from approximately 1 to 46% of the dry weight of the plant, depending on the time of year. In winter the soil content was consistently greater than 23% of plant dry weight (Beresford and Howard 1991). Two other studies that measured the soil content of harvested cattle feed found that soil contributed less than 1% of the dry weight of the feed (Fries et al. 1981; Zach and Mayoh 1984). It is reasonable to assume that forage is not washed before feeding animals under normal conditions. However, many of the plant crop studies and one of the two studies of forage crops used experimental methods that either protected the leaves from contact with soil or washed it away after harvesting. Thus, the contribution of contaminated soil to harvested forage crop PCDD/F contamination may not have been adequately assessed by the studies to date. More evidence is needed to evaluate this potentially important contributor to animal uptake of PCDD/Fs.

### Animal Foods

The results of this review indicate that consumption of contaminated feed or grazing of cattle on treated land is likely to increase the PCDD/F levels in meat products. Unlike the plant studies, most of the studies examining the impact of PCDD/F contamination on animal tissue used feed contaminated at levels low enough that they might be encountered in practice.

The relationship between feed contamination levels and concentrations in the fatty tissue of cattle (Figure 2, Table 7) is considerably stronger than that for plant tissues, with a coefficient two to three orders of magnitude higher than for most plants and one order higher than for the family Cucurbitaceae. The coefficient of the relationship is greater than 1, suggesting bioaccumulation. As an example, the PCDD/F concentration in beef tissue may increase by up to 10 pg TEQ/g fat at the relatively low contamination level of 5 pg TEQ/g in feed (Table 7). This suggests that the use of dioxin/furan-contaminated sewage sludge on grazing land or on land used to grow cattle feed may result in increased human exposure to PCDD/Fs through the diet, especially if the sludge is highly contaminated.

There were insufficient data to conclude whether consumption of feed grown on land treated with sewage sludge or grazing of animals on sludge-amended land is likely to increase the PCDD/F levels in milk products. Few studies examined the relationships between contamination of feed or grazing land and milk contamination from cows (Fries et al. 1999; Jilg et al. 1992; McLachlan et al. 1990, 1994; McLachlan and Richter 1998), and no clear relationship could be seen in the data. Overall, the studies that examined the relationship between feed or soil PCDD/F concentration and milk concentration show that PCDD/Fs are excreted in milk. The amount excreted appears to be dependent on the timing of PCDD/F contamination in the diet (Jilg et al. 1992; Jones et al. 1989). There may be only a minimal impact of sewage sludge use on milk, especially if a sufficient time lag is provided between sludge application and milking for human consumption. However, the data are still very limited.

The application of sewage sludge to grazing or forage land presents additional exposure risk to animals beyond that resulting from direct uptake of PCDD/Fs by the crops. Animals consume soil along with fodder, either by eating the soil directly while grazing or by consuming plants (e.g., grass, hay, or beetroot) to which soil has adhered (McLachlan et al. 1996a; Zach and Mayoh 1984). As a result, they may directly ingest sludge that has been applied to pastureland. Although estimates vary, cattle, sheep, and swine may consume an average of 6–7% (up to 18% during seasons of sparse forage) of their ingested dry matter as soil (Fries 1996; Pohl et al. 1995). Studies from the Netherlands and the United States, where grazing is seasonal and cattle are given plenty of supplemental feed, suggest that cows may ingest an average of 150–300 g of soil per day (1–2% of their dry matter intake) (McLachlan et al. 1996a). At a worst-case estimate of 30 pg TEQ/g soil, this would correspond to an additional intake of up to 9 ng PCDD/F per cow per day. Based on an analysis of studies from New Zealand, the United Kingdom, and the United States, Fries (1996) estimated that a 500-kg dairy cow would ingest 900 g of soil per day. With a PCDD/F concentration of 30 pg TEQ/g soil, this would contribute 27 ng PCDD/F per cow per day.

### Limitations

One of the primary limitations of this review is the small number of studies relevant to the subject at hand. All the data related to plant foods were taken from only six articles, and the variety of plant species represented is quite small relative to the number of food crops that could potentially be exposed to recycled sewage sludge. No studies were identified that measured PCDD/Fs in animals other than cattle fed from sludge-amended land. Although there were eight articles reporting background concentrations of PCDD/F in animal tissue, the level of PCDD/F contamination in the feed or grazing land of these animals was not reported.

There were no field-based plant studies and few animal uptake studies that examined the effects of real sludge application practices. This is especially important with respect to harvested forage crops, for which the contribution of soil adherence is not known.

Many studies did not describe the details of the analytical methods used (including limits of detection) or state whether crop samples were washed before analysis. Field practices such as sludge application rate, application method, PCDD/F concentration, and fertilization/harvesting time may influence the uptake of PCDD/F. Unfortunately, such factors could not be considered in this review because the information was not usually reported in the published studies. Furthermore, although the TEQ system is useful when comparing samples with differing congener profiles, it is somewhat limited in that any differences in uptake or behavior of individual congeners is not taken into account.

### Gaps in the Published Research

Although there is some empirical evidence to suggest that there is an impact of sewage sludge application on PCDD/F uptake by grazing animals but minimal uptake from sludge to plants, there are a number of significant gaps in the data. Controlled field studies are needed that include variables such as application rate, timing, and method and that assess crops and animals exposed under realistic conditions. Repeat studies must be conducted to determine the reliability of the data, and more species need to be assessed. It is essential that the complex issue of additional animal exposure to sewage sludge through soil consumption or adherence to forage crops be examined. Information is also needed on the effects on animals other than cows, for example, swine and poultry.

## Conclusions

The results reported here, based on published empirical data, were compared with the results of studies that used pathway modeling to predict the effect of land application of sewage sludge on PCDD/F contamination in food and were similar. Investigators using models have concluded that *a*) sewage sludge application may lead to slight increases in PCDD/F concentration in the peel of root crops (Duarte-Davidson and Jones 1996; Jackson and Eduljee 1994; Wild and Jones 1992) or in members of the Cucurbitaceae family (Jones and Sewart 1997), but would have a negligible impact on other aboveground plants (Duarte-Davidson and Jones 1996; Jones and Sewart 1997; Rappe et al. 1999; Wild and Jones 1992; and that *b*) sewage sludge application on grazing or forage land could significantly increase human dietary exposure to PCDD/F (Duarte-Davidson and Jones 1996; Jackson and Eduljee 1994; Jones and Sewart 1997; McLachlan et al. 1996a; Rappe et al. 1999; Wild and Jones 1992; Wild et al. 1994). A recent human health risk assessment (U.S. EPA 2004) found that land application of sewage sludge would lead to a negligible increase in cancer cases even among the most highly exposed groups. Noncancer health risks were not assessed. Our review examined the potential for increased human foodborne exposure rather than potential health outcomes.

In conclusion, the available empirical evidence indicates that application of sewage sludge to agricultural land may have a small impact on the levels of PCDD/F found in root vegetables, aboveground plant foods, and forage crops. The impact in animal tissues is likely to be considerably greater. Therefore, before sludge application, careful consideration should be given to the types of agricultural products grown. Minimizing the PCDD/F content would also reduce human exposure potential in land application of sewage sludge.

## Figures and Tables

**Table 1:** Concentrations of PCDD/F in sewage sludge, sorted by country and year.

Reference	Country	Year	Source of material	*n*	Mean concentration (pg TEQ/g)	Range (pg TEQ/g)
Ho and Clement 1990	Canada	1986	Treated municipal sludge	50	NA	0.0005–0.0015
			Raw municipal sludge	50	NA	0.0026–0.0051
van Oostdam and Ward 1995	Canada	1990–1993	Primary sludge	4	16.6 (dw)	2.3–49.6
Healey and Bright 2000	Canada	1998–1999	Municipal wastewater treatment plants	26	40 (dw)	5.6–250
Lamparski et al. 1984	USA	1933	Treated municipal sludge	1	87.7 (dw)	
		1981	Treated municipal sludge	1	88.9 (dw)	
		1982	Treated municipal sludge	1	80.8 (dw)	
Telliard et al. 1990	USA	1988–1989	Public-owned sewage treatment works	211	38.38 (ww)	0.039–1252.9
Malloy et al. 1993	USA	1990–1992	Municipal yard waste compost	11	29.6	5–91
			Municipal solid waste compost	6	46.5	19–96
			Municipal solid waste + dewatered sewage sludge compost	4	56	37–87
Wilson et al. 1997	U.K.	NA	Anaerobically digested sewage sludge	1	19 (dw)	
McLachlan et al. 1996b	U.K.	1968	Rural uncontaminated sewage sludge	2	230 (dw)	200–280
Sewart et al. 1995	U.K.	1992	Digested sludges from sewage treatment plants	8	72 (dw)	19–206
		1942–1960	Archived samples from 1942 to 1960	7	148 (dw)	18–402
Rappe et al. 1989	Sweden	NA	Urban sludge	1	23.9	
			Rural sludge	1	23.1	
Naf and Broman 1990	Sweden	May–Aug 1989	Anaerobically digested sludge from urban wastewater treatment plant	1	31 (dw)	
Broman et al. 1990	Sweden	May–Aug. 1989	Digested and dewatered sludge	4	79 (ow)	41–130
Grossi et al. 1998	Brazil	1990–?	Municipal solid waste compost from the following:			
			Urban	11	57 (dw)	11–150
			Small cities	5	27 (dw)	3–163
			Coastal sandy	3	8 (dw)	5–11
			New, some industrial waste	2	54 (dw)	10–99
Disse et al. 1995	Germany	NA	Undigested sludge from rural area	1	9 (dw)	
			Undigested sludge from municipal area with no heavy industry	1	20 (dw)	
			Undigested sludge from municipal area with metal industry	1	200 (dw)	
McLachlan and Reissinger 1990	Germany	NA	Local wastewater treatment plant	1	42 (dw)	
Horstmann et al. 1992	Germany	1991	Anaerobically digested sewage sludge	1	48 (dw)	
			Primary sludge (dry conditions)	9	31.4 (dw)	15–64
			Primary sludge (rainy conditions)	2	28.5 (dw)	21–36
Eljarrat et al. 1999	Spain	1994–1998	Sludges from rural, urban, and industrial wastewater treatment plants	19	55 (dw)	7–160
		1979–1987	Archived samples from 1979 to 1987	24	620 (11.3-fold increase)	29–8,300
Molina et al. 2000	Spain	NA	Aerobic sewage treatment plant	1	68.1 (dw)	
Eljarrat et al. 1997	Spain	1986, 1987	Sludge from urban wastewater treatment plants (aerobic digestion)	7	144 (dw)	74–260

Abbreviations: dw, dry weight; NA, no data available; ow, organic weight; ww , wet weight.

**Table 2:** Concentrations of PCDD/F in soil (background and sludge amended), sorted by year of publication.

Reference	Country	Year	Source of material	Sludge concentration (pg TEQ/g)	*n*	Mean concentration (pg TEQ/g)	Range (pg TEQ/g)
Creaser et al. 1989	U.K.		Soil at intersection points of a 50-km grid	NA	77	23.4 (dw)	1.2–161.9
Broman et al. 1990	Sweden	1989	Agricultural land near major roads	NA	4	29 (ow)	13–49
			Agricultural land not near major roads	NA	4	17 (ow)	9–32
McLachlan and Reissinger 1990	Germany		Farmland	NA	1	0.84 (dw)	
			Farmland	42 (dw)	2	6.55 (dw)	3.7–9.4
			Meadow	42 (dw)	1	15 (dw)	
Kjeller et al. 1991	U.K.	1986	Semirural experimental plots	NA	3	1.4 (dw)	
Sund et al. 1993	Australia	1990	Soil from urban and industrial areas	NA	7	2.3	0.09–8.2
van Oostdam and Ward 1995	Canada	1990–1993	Background soil	NA	53	5.0 (dw)	ND–57
McLachlan et al. 1996b	U.K.	1968, 1972, 1976, 1981, 1985, 1990	Experimental agricultural land	NA	6	1.3 (dw)	0.88–2.0
			Sludge applied experimentally in 1968	230 (dw)	5	8.8 (dw)	6.5–13
Eljarrat et al. 1997	Spain	1986–1987	Acidic and basic agricultural soil	NA	2	1.7 (dw)	0.3–3.1
			Urban wastewater treatment plants (aerobic digestion)	144 (dw)	4	4.6 (dw)	2.4–8.6
Wilson et al. 1997	U.K.		Plowed plot	NA	4	2.0 (dw)	1.8–2.2
			Pasture plot	NA	4	1.9 (dw)	1.7–2.0
			Plowed plot (15–20 cm)	19	4	2.7 (dw)	2.4–3.0
			Pasture plot (surface application)	19	4	2.8 (dw)	1.6–4.3
Molina et al. 2000	Spain		Alkaline soil	NA	2	0.37 (dw)	0.34–0.39
			7.5% sludge (time 0)	68.1 (dw)	1	2.43 (dw)	
			7.5% sludge (1 year)	68.1 (dw)	1	2.37 (dw)	
			15% sludge (time 0)	68.1 (dw)	1	5.28 (dw)	
			15% sludge (1 year)	68.1 (dw)	1	4.61 (dw)	
			Quarry	NA	2	0.84 dw)	0.76–0.92
			Direct application of 7.5% sludge (time 0)	68.1 (dw)	1	1.4 (dw)	
			Direct application of 7.5% sludge (4 years)	68.1 (dw)	1	12.1 (dw)	
			Soil–sludge mixture 7.5% (time 0)	68.1 (dw)	1	3.14 (dw)	
			Soil–sludge mixture 7.5% (4 years)	68.1 (dw)	1	4.24 (dw)	
			Direct application of 15% sludge (time 0)	68.1 (dw)	1	5.26 (dw)	
			Direct application of 15% sludge (4 years)	68.1 (dw)	1	8.50 (dw)	
			Soil–sludge mixture 15% (time 0)	68.1 (dw)	1	2.56 (dw)	
			Soil–sludge mixture 15% (4 years)	68.1 (dw)	1	4.24 (dw)	

Abbreviations: dw, dry weight; NA, no data available; ND, not detected; ow, organic weight.

**Table 3:** PCDD/F concentrations in root vegetables, sorted by year of publication.

Reference	Growing environment	Source of PCDD/F	Soil concentration (pg TEQ/g)	Plant type (part)	*n*	Mean plant concentration (pg TEQ/g) (dw)	Range of plant concentration (pg TEQ/g) (dw)
Prinz et al. 1991	Field conditions	None	68 (dw)	Potato (tuber)	2	~ 0.5	
		Incinerator	274 (dw)	Potato (tuber)	2	< LOD	
		Incinerator	670 (dw)	Potato (tuber)	2	~ 0.6	
		Incinerator	788 (dw)	Potato (tuber)	2	~ 0.3	
		None	68 (dw)	Carrot (root)	2	~ 0.6	
		Incinerator	274 (dw)	Carrot (root)	2	~ 0.6	
		Incinerator	670 (dw)	Carrot (root)	2	~ 2.8	
		Incinerator	788 (dw)	Carrot (root)	2	~ 2.0	
		Incinerator	670 (dw)	Celery	2	~ 0.4	
		Incinerator	788 (dw)	Red beet (tuber)	2	~ 0.4	
Hulster and Marschner 1993	Field conditions	None	4.8	Potato (unpeeled)	NA	~ 0.2	
		Incinerator	328	Potato (unpeeled)	NA	~ 0.6	
			845	Potato (unpeeled)	NA	~ 1.2	
			2,390	Potato (unpeeled)	NA	~ 1.6	
		None	4.8	Potato (peeled)	NA	~ 0.1	
		Incinerator	328	Potato (peeled)	NA	~ 0.1	
			845	Potato (peeled)	NA	~ 0.1	
			2,390	Potato (peeled)	NA	~ 0.1	
Schroll and Scheunert 1993	Closed system	None	0	Carrots (roots)	2	< LOD	
	Growing chamber	OCDD added to soil	6,400 (dw)	Carrots (roots)	2	4,811.1 397.8 (fw)	3134.3–6488.5 259.1–536.4 (fw)
Muller et al. 1994	Field conditions	None	5 (dw)	Carrots (peel)	1	0.55	
		Incinerator	56 (dw)	Carrots (peel)	2	3.08	2.86–3.3
		None	5 (dw)	Carrots (cortex)	1	0.27	
		Incinerator	56 (dw)	Carrots (cortex)	2	0.29	0.28–0.3
		None	5 (dw)	Carrots (stele)	1	0.32	
		Incinerator	56 (dw)	Carrots (stele)	2	0.395	0.29–0.5
		None	5 (dw)	Carrots (whole)	1	0.35	
		Incinerator	56 (dw)	Carrots (whole)	2	0.96	0.87–1.05

Abbreviations: dw, dry weight; fw, fresh weight; LOD, limit of detection; NA, no data available.

**Table 4:** PCDD/F concentrations in crops with edible parts grown aboveground, sorted by year of publication.

Reference	Growing environment	Source of PCDD/F	Soil concentration (pg TEQ/g)	Plant type (part)	*n*	Mean plant concentration (pg TEQ/g dw)	Range of plant concentration (pg TEQ/g dw)
Prinz et al. 1991	Field conditions	None	68 (dw)	Salad	2	~ 0.4	
		Incinerator	200 (dw)	Salad	2	~ 3.2	
		Incinerator	274 (dw)	Salad	2	~ 4.3	
		Incinerator	670 (dw)	Salad	2	~ 9.2	
		Incinerator	788 (dw)	Salad	2	~ 6.6	
		None	68 (dw)	Silver beet	2	~ 0.3	
		Incinerator	25 (dw)	Silver beet	2	~ 3.5	
		Incinerator	670 (dw)	Silver beet	2	~ 9.8	
		Incinerator	788 (dw)	Silver beet	2	~ 7.0	
		Incinerator	199 (dw)	Kale	2	~ 7.3	
		Incinerator	200 (dw)	Kale	2	~ 6.6	
		Incinerator	274 (dw)	Kale	2	~ 6.3	
		Incinerator	788 (dw)	Kale	2	~ 2.0	
		Incinerator	274 (dw)	Endive	2	~ 2.5	
		Incinerator	788 (dw)	Endive	2	~ 17.8	
		Incinerator	670 (dw)	Leek	2	~ 1.6	
		Incinerator	670 (dw)	Cucumber	2	~ 0.8	
		Incinerator	670 (dw)	Bean	2	~ 0.6	
		Incinerator	788 (dw)	Kohlrabi	2	~ 0.3	
		Incinerator	788 (dw)	Savoy	2	~ 0.5	
Hulster and Marschner 1993	Field conditions	None	4.8	Lettuce leaves	NA	~ 0.2	
		Incinerator	845	Lettuce leaves	NA	~ 0.3	
		Incinerator	328	Lettuce leaves	NA	~ 1.3	
		None	4.8	Lettuce (whole)	NA	~ 0.2	
		Incinerator	845	Lettuce (whole)	NA	~ 0.4	
		Incinerator	328	Lettuce (whole)	NA	~ 1.4	
Schroll and Scheunert 1993	Closed system	Treated soil	6,400 (dw)	Carrots (stem)	2	2306.2	2029.4–2582.9
Hulster et al. 1994	Field conditions	None	0.4 (dw)	Zucchini (fruit)	2	1.0	0.9–1.1
			0.4 (dw)	Zucchini (fruit)	2	0.6	0.5–0.7
		Chlorine–alkaline–	148 (dw)	Zucchini (fruit)	2	20.0	19.1–21.0
		electrolysis residues	148 (dw)	Zucchini (no soil–fruit contact)	2	20.5	19.4–21.6
			328 (dw)	Zucchini (fruit)	2	17.2	17.0–17.4
			2,390 (dw)	Zucchini (fruit)	2	54.9	54.6–55.2
		Chlorine–alkaline–	148 (dw)	Pumpkin (outer fruit)	2	11.8	11.6–12.0
		electrolysis residues	148 (dw)	Pumpkin (inner fruit)	2	3.25	3.1–3.4
			148 (dw)	Cucumber (outer fruit)	2	2.35	2.3–2.4
			148 (dw)	Cucumber (inner fruit)	2	0.2	0.2–0.2
Muller et al. 1994	Field conditions	None	5 (dw)	Peas (pods)	1	0.13	
		Incinerator	56 (dw)	Peas (pods)	1	0.12	
		None	5 (dw)	Peas (seeds)	1	< 0.01	
		Incinerator	56 (dw)	Peas (seeds)	1	0.04	
		None	5 (dw)	Peas (whole)	1	0.08	
		Incinerator	56 (dw)	Peas (whole)	1	0.09	
		None	5 (dw)	Lettuce (outer leaves)	1	0.13	
		Incinerator	56 (dw)	Lettuce (whole)	2	0.21	0.21–0.21

Abbreviations: dw, dry weight; NA, no data available.

**Table 5:** PCDD/F concentrations in tree fruits, sorted by year of publication.

Reference	Growing environment	Source of PCDD/F	Soil concentration (pg TEQ/g) (dw)	Plant type (part)	*n*	Mean plant concentration (pg TEQ/g)	Range of plant concentration (pg TEQ/g)
Prinz et al. 1991	Field conditions	Incinerator	670	Plum	2	~ 1.1 (dw)	
				Strawberry	2	~ 0.8 (dw)	
				Apple	2	~ 1.4 (dw)	
Muller et al. 1993	Field conditions	Chlorine–alkaline–electrolysis residues	48 (subsoil)	Pear 2 (washed, whole)	1	25 (fw)	
			14,530 (subsoil)	Pear 1 (unprocessed, whole)	2	33 (fw)	20–46
				Pear 1 (washed, peel)	2	123.5 (fw)	105–142
				Pear 1 (washed, pulp)	2	15 (fw)	8–22
				Pear 1 (washed, whole)	2	36 (fw)	27–45
				Pear 1 (wrapped, whole)	2	14 (fw)	11–17
			1,950 (subsoil)	Apple (washed, pulp)	1	8 (fw)	
				Apple (washed, peel)	1	46 (fw)	
				Apple (washed, whole)	1	14 (fw)	

Abbreviations: dw, dry weight; fw, fresh weight.

**Table 6:** PCDD/F concentrations in forage crops.

Reference	Growing environment	Source of PCDD/F	Soil concentration (pg TEQ/g)	Plant type (part)	*n*	Mean plant concentration (pg TEQ/g dw)
Hulster and Marschner 1993	Field conditions	None	4.8	Hay	NA	~ 1
		Incinerator	328	Hay	NA	~ 4
		Incinerator	845	Hay	NA	~ 3
		Incinerator	2,390	Hay	NA	~ 10
		Incinerator	5,752	Hay	NA	~ 6
		None	4.8	Herbs (hay)	NA	< LOD
		Incinerator	328	Herbs (hay)	NA	~ 0.5
		Incinerator	845	Herbs (hay)	NA	~ 0.7
		Incinerator	2,390	Herbs (hay)	NA	~ 0.8
		Incinerator	5,752	Herbs (hay)	NA	~ 0.9
		None	4.8	Grass (hay)	NA	< LOD
		Incinerator	328	Grass (hay)	NA	~ 0.1
		Incinerator	845	Grass (hay)	NA	~ 0.2
		Incinerator	2,390	Grass (hay)	NA	~ 0.1
		Incinerator	5,752	Grass (hay)	NA	~ 0.2

Abbreviations: dw, dry weight; LOD, limit of detection; NA, no data available.

**Table 7:** Mean projected increase in concentration of PCDD/F in food with a given increase in soil or feed concentration.

Food type		Increase in soil or feed PCDD/F concentration (pg TEQ/g dw)*^a^*				
		1*^b^*	5	10	15	30
	*n*	Projected increase in food concentration (pg TEQ/g dw)				
Herbs	5	0.0001 (0.00006)*^c^*	0.00 (0.00)*^d^*	0.00 (0.00)*^d^*	0.00 (0.00)*^d^*	0.00 (0.00)*^d^*
Potato tuber	9	0.0004* (0.000063)	0.00 (0.00)	0.00 (0.01)	0.01 (0.01)	0.01 (0.02)
Hay	5	0.0008 (0.000703)	0.00 (0.01)	0.00 (0.02)	0.00 (0.03)	0.00 (0.06)
Tree fruits (fw)	9	0.0016 (0.00185)	0.01 (0.02)	0.02 (0.05)	0.02 (0.07)	0.05 (0.15)
Carrot root	13	0.0027* (0.000608)	0.01 (0.01)	0.03 (0.02)	0.04 (0.05)	0.08 (0.11)
Leafy vegetables	26	0.0042 (0.00255)	0.01 (0.04)	0.03 (0.09)	0.06 (0.13)	0.12 (0.21)
Cucurbitaceae	9	0.019* (0.00503)	0.07 (0.12)	0.17 (0.26)	0.27 (0.41)	0.55 (0.84)
Animal tissue	18	1.458* (0.278)5.80 (8.00)	13.1 (18.0)	21.9 (28.0)	47.4 (58.0)	

**a**Soil/feed concentration values are intended to represent the following potential scenarios: 0–1 pg TEQ/g represents the likely concentrations found in forage crops grown in soil with minimal background PCDD/F contamination (Hulster and Marschner 1993); 0.1–4 pg TEQ/g represents the likely concentrations found in forage grown in sludge-amended soil; 1–10 pg TEQ/g is the typical range in sludge-amended agricultural soil; and the concentrations found in forage grown in highly contaminated soil (> 670 pg TEQ/g) (Hulster and Marschner 1993; Prinz et al. 1991); 15 pg TEQ/g represents the maximum concentration reported in sludge-amended soil (McLachlan and Reissinger 1990); 30 pg TEQ/g represents the maximum mean concentration reported in soil (not sludge amended) (Broman et al. 1990).

**b**Coefficient of relationship between food concentration and soil or feed concentration.

**c**Values in parentheses are standard error of the coefficient.

**d**Values in parentheses are upper 95% confidence limits of the increase in food concentration.

*Regression coefficient significant at *p* < 0.05.

**Table 8:** Concentrations of PCDD/F in food from cattle, sorted by year of publication.

Reference	Source of PCDD/F	Feeding time	Mean food concentration (pg TEQ/g)	Tissue	No. of animals	Mean tissue concentration (pg TEQ/g fat)	Range of tissue concentration (pg TEQ/g)
Jensen et al. 1981	Experimental	28 days	24 ± 5	Fat	7	84	66–95
				Liver	7	8.2	7–10
				Kidney	7	7	6–8
				Muscle	7	2	2
Jones et al. 1989	Single oral dose in grain	1 dose	~ 3557	Fat	2	105	80–130
	Single oral dose in soil	1 dose	~ 3557	Fat	2	155	130–180
McLachlan et al. 1990	None	NA	6.9	Milk	1	1.39	
Jilg et al. 1992	Hay grown in contaminated soil (1,944 pg TEQ/g dw)	19 weeks	2 (range 0.5–8.7)	Plasma	4	1.95	0.8–4.1
				Fat	4	1.1	0.6–2.8
				Muscle	4	1.75	1.3–2.8
				Milk (weeks 1–19)	4	1.88	0.8–3.0
				Milk (weeks 20–28)	3	1.13	0.6–2.1
McLachlan et al. 1994	None	6 months	0.19 (dw)	Milk	12	0.9	
	None	6 months	0.22 (dw)	Milk	12	1.3	
	Silage from sludge- treated land	6 months	0.35 (dw)	Milk	12	1.2	
	Silage from sludge- treated land	6 months	1.2 (dw)	Milk	12	2.3	
Schecter et al. 1994	None		NA	Beef	4	0.578 (ww)	0.04–1.5 (ww)
			NA	Dairy	5	0.348 (ww)	0.04–0.7 (ww)
Winters et al. 1996	None		NA	Back fat	63	0.35 (SE 0.08)	< LOD–3.8
Fiedler et al. 1997	None		NA	Fat	3	0.67 ± 0.17	0.528–1.1
			NA	Dairy fat	9	0.77 ± 0.10	0.416–0.970
Feil and Ellis 1998	None		NA	Perirenal fat	20	4.1275 (ww)	0.3341–30.8373
McLachlan and Richter 1998	None	12 weeks	0.2 (dw)	Milk (whole)	4	0.015 (whole milk)	0.010–0.02 (whole milk)
	Silage from sludge- treated land	17 days	3.2 (dw)	Milk (whole)	4	0.049 (day 23)	0.031–0.069 (day 23)
Fries et al. 1999	None		NA	Milk	4	0.315	
	PCP-treated wood	58 days	0.289 (dw)	Milk	4	5.518	
Richter and McLachlan 2001	None	10 weeks	0.2 (dw)	Muscle	2	0.41	0.30–0.51
				Fat	2	0.47	0.34–0.61
				Liver	2	6.5	5.1–7.9
				Kidney	2	0.50	0.41–0.58
	Silage from sludge- treated land	17 days	3.2 (dw)	Muscle	2	0.70	0.54–0.91
				Fat	2	0.64	0.49–0.79
				Liver	2	20.5	17.0–24.0
				Kidney	2	0.74	0.61–0.86
Thorpe et al. 2001	None (testing at 31 weeks)	28 days	NA	Liver	4	3.9	
				Muscle	4	5.9	
				Fat	4	3.7	
	Prepared pellets (testing at 31 weeks)	28 days	~ 41.3 (330,000 pg TEQ/day)	Liver	4	118.5	
				Muscle	4	57.3	
				Fat	4	27.2	

Abbreviations: dw, dry weight; LOD, limit of detection; NA, no data available; PCP, pentachlorophenol.
